# Biochar production increases the polycyclic aromatic hydrocarbon content in surrounding soils and potential cancer risk

**DOI:** 10.1007/s11356-013-2334-1

**Published:** 2013-11-26

**Authors:** Marcin Kuśmierz, Patryk Oleszczuk

**Affiliations:** Department of Environmental Chemistry, Faculty of Chemistry, 3 Maria Curie-Skłodowska Square, Lublin, 20-031 Poland

**Keywords:** PAHs, Biochar, Soil, Risk assessment, Molecular diagnostic ratios, Incremental lifetime cancer risks

## Abstract

The objectives of the study were the identification of the source of contamination of soils and estimation of the potential cancer risk that may be caused by contact with soils situated in the vicinity of biochar production sites. Samples of soils collected in the immediate vicinity of traditional biochar-producing plants, located within the area of the Bieszczady National Park (Poland), were analysed for the content of polycyclic aromatic hydrocarbons (PAHs). The sum of the content of 16 PAHs varied within the range of 1.80–101.3 μg/g, exceeding the norms permitted in many European countries. The calculated coefficients on the basis of which one can determine the origin of PAHs (molecular diagnostic ratios) demonstrated that the potential source of PAHs in the soils may be processes related with the production of biochar. Estimation on the basis of the results of incremental lifetime cancer risks (ILCRs) within the range of 2.33 · 10^−4^–1.05 · 10^−1^ indicated that the soils studied may constitute a significant cancer risk for persons who have contact with them. The values of ILCRS should be considered as at least high, which permits the conclusion that sites of that type may create a hazard to human health.

**Electronic supplementary material:**

The online version of this article (doi:10.1007/s11356-013-2334-1) contains supplementary material, which is available to authorized users.

## Introduction

The method of sequestration (capture and long-term storage) of atmospheric CO_2_ proposed by Lehmann (2007), consisting in the transformation of biomass into biochar and its deposition in soils, gained notable interest in the world of science. Research in this area is conducted in many places in the world, e.g. in Zambia, Tanzania, Malaysia and Nepal. Biochars used in such studies are usually produced locally, with traditional methods. Also, the unloading of kilns, reloading, transport and dosage of biochars to soils are most frequently done by hand. Unfortunately, due to their properties, biochars may be dangerous to ecosystems and to human health. Polycylic aromatic hydrocarbons (PAHs) contained in biochars create a risk to living organisms and to humans when they come in contact with those materials as well as with the soils amended with them (Oleszczuk et al. 2013; Sims and Overcash 1983). Workers employed in the production and transport of biochars are particularly exposed. The area of that risk increases following the growing popularity of biochar use.

The methods of biochar production are simple, known for a very long time, and can be applied in countries that do not have advanced technologies. The results of numerous studies indicate a positive effect of biochars on the physical, chemical and biological properties of soils. Biochars are becoming a material that is more and more often used for the improvement of soil properties, with simultaneous beneficial effect consisting in mitigating climate change. With relation to the growing popularity of the utilisation of biochar, an increasing number of people will have contact with biochars and with soils remediated with their use, which may cause a notable expansion of the risk group. This results from the fact that in the course of biochar production, highly dangerous PAHs are formed (Freddo et al. 2012; Oleszczuk et al. 2013). They are formed through the degradation of lignins and cellulose, on the pathway of unimolecular reactions, such as dealkylation, dehydrogenation, cyclisation, aromatisation and/or radical reactions. Sixteen of them, due to their potentially mutagenic and carcinogenic properties, have been given the status of priority substances in the USA and in the European Union (Sims and Overcash 1983).

Biochar produced with the traditional method is characterised by very low mechanical strength and high brittleness; therefore, during the emptying of kilns, shifting, reloading and transport, it undergoes considerable fragmentation. As a consequence, in the vicinity of kilns, fine coal gets into the soil, and the silt fractions of biochar are carried with the wind over longer distances. In the immediate vicinity of the kilns, soil may also be contaminated with the liquid products of pyrolysis. PAHs migrating into the soil together with biochar are very hard-biodegradable (Koelmans et al. 2006), which contributes to their increased stability in the soils and extends the time over which they may pose a threat to organisms and to the environment.

Workers employed in the production and transport of biochar are particularly exposed to contact with contaminated soil. Soil particles cannot only settle on the skin but they can also be accidentally ingested and inhaled. As mentioned earlier, the numbers of people involved in the production of biochar, and thus exposed to contact with contaminated soil, will grow following the scale of application of those materials. In this context, the assessment of risk related with environmental pollution resulting from biochar production is important.

The objectives of the study were to determine the level of polycyclic aromatic hydrocarbons in soils in the vicinity of traditional biochar kilns, to identify—by means of the molecular diagnostic ratios (MDRs)—the sources of the PAHs and to assess the risk of cancer related with the presence of those compounds in soils. This will permit to determine how sites related with the production of biochar affect the quality of soils and—indirectly—human health.

## Methods and materials

### Soil sampling and preparation

Soil samples for analyses were taken from five localities where biochars are produced with the traditional methods on a seasonal basis: Smerek (W1a, W1b, W1c), Habkowice (W2a, W2b), Smolnik (W2a, W3b), Maniów (W4a, W4b) and Muczne (W5a, W5b). All of those localities are situated in the area on the Bieszczady National Park in the south-eastern part of Poland (Fig. S1). The portable ring kilns with a capacity of 15 m^3^ are used to obtain biochars in this area. Mixture of grey alder, silver birch and aspen poplar is used for biochar production. During the burning process, the temperature inside the kiln is about 400–500 °C. The primary products of the process are solids (charcoal, coke breeze), liquids (tar, methanol, water), gases and atmospheric particles bound with organic and inorganic contaminants. All the biochar-producing facilities from which soil samples were taken were situated in forest areas.

Soil samples were collected in the spring of 2012 from a 0- to 20-cm horizon close to the kilns (5–10 m) using a stainless steel corer (5 × 60 cm i.d.). The cores were placed into ziplock bags and transported to the laboratory. Samples for the determination of physico-chemical properties and PAH contents were air-dried in an air-conditioned storage room for 2 days (20 °C, in darkness), mechanically crushed and passed through a 2-mm sieve. Then, samples were kept in glass jars (previously cleaned by rinsing with acetonitrile) and stored in a laboratory freezer (−4 °C). Physico-chemical properties of the soil studied are presented in Table S1 (Supplemental Material, page 3).

### PAH analysis

Dry soil samples were extracted using an accelerated solvent extractor (ASE 100) from Dionex GmbH (Idstein, Germany). The extraction program was based on the ASE Dionex application note 313 for PAHs in soil and sediment. Next, the extracts were evaporated and purified by solid-phase extraction according to the procedure described elsewhere (Oleszczuk and Baran 2004). A qualitative and quantitative analysis of PAHs was carried out on a high-performance liquid chromatograph (Waters, e2695) with photodiode array (Waters 2998) and fluorescence (Waters 2475) detectors. A Waters PAH C18RP (5 μm, 4.6 × 250 mm) column was used for the separation of 16 PAHs. Detection was carried out at 254 nm. Elution of all PAH was carried out for 32 min. Recoveries for the total procedures (sample preparation, extraction and SPE) ranged between 81 and 90 % for individual PAHs. Precision expressed as relative standard deviation was below 12 %. The concentrations reported here have, therefore, not been corrected for losses. The procedural blank was determined by going through the same extraction and clean-up procedures for each series of samples. None of the analytical blanks were found to have detectable contamination of the monitoring PAHs, and thus, the results were not blank-corrected.

A diagnostic tool that is frequently used for the identification of the sources of PAHs is the MDRs (Oleszczuk and Pranagal 2007; Tobiszewski and Namieśnik 2012). Their application is based on the assumption that certain PAHs are emitted at relatively constant source-related proportions and that those proportions are retained after reaching the receiver (Katsoyiannis et al. 2011).

Individual MDRs do not uniquely identify the source of PAHs: some of them, like, e.g. BaA/(BaA+CHR), are characterised by considerable variability within a source type; others, like FLA/(FLA+PYR), may have similar values for various sources (Tobiszewski and Namieśnik 2012). For a more accurate determination of the origin of PAHs, two or more MDRs can be used. In this study, three MDRs were used: FLA/(FLA+PYR), IcdP/(IcdP+BghiP) and ANT/(ANT+PHE). However, some authors question the applicability of the latter ratio (Brändli et al. 2008); their use is widely adopted in the literature for the identification of the origin of PAHs in soils (Bucheli et al. 2004; De La Torre-Roche et al. 2009; Liu et al. 2010; Maliszewska-Kordybach et al. 2008; Marusenko et al. 2011; Plachá et al. 2009; Wang et al. 2007, 2010).

### Incremental lifetime cancer risk

Assessing the threat to human health, the incremental lifetime cancer risks (ILCRs) were estimated. It was assumed that PAHs penetrate into the human organism in three ways: through accidental ingestion of soil particles, inhalation of soil particles and dermal contact. The calculations were performed on the basis of the following equations (Peng et al. 2011; US EPA, OSWER 1991, 2009):

1 $$ \mathrm{CS}={\mathrm{C}}_{\mathrm{NAP}}\cdot {\mathrm{TEF}}_{\mathrm{NAP}}+\dots +{\mathrm{C}}_{\mathrm{IcdP}}\cdot {\mathrm{TEF}}_{\mathrm{IcdP}} $$

2 $$ {\mathrm{ILCRs}}_{\mathrm{ing}\mathrm{estion}}=\left(\mathrm{CS}\cdot \mathrm{CF}\cdot {\mathrm{CSF}}_{\mathrm{ing}}\cdot {\left(\mathrm{BW}/70\right)}^{1/3}\cdot {\mathrm{IR}}_{\mathrm{soil}}\cdot \mathrm{EF}\cdot \mathrm{ED}\right)/\left(\mathrm{BW}\cdot \mathrm{AT}\right) $$

3 $$ {\mathrm{ILCRs}}_{\mathrm{der}\mathrm{nal}}=\left(\mathrm{CS}\cdot \mathrm{CF}\cdot {\mathrm{CSF}}_{\mathrm{der}}\cdot {\left(\mathrm{BW}/70\right)}^{1/3}\cdot \mathrm{SA}\cdot \mathrm{EV}\cdot \mathrm{AF}\cdot \mathrm{ABS}\cdot \mathrm{EF}\cdot \mathrm{ED}\right)/\left(\mathrm{BW}\cdot \mathrm{AT}\right) $$

4 $$ {\mathrm{ILCRs}}_{\mathrm{inh}\mathrm{alation}}=\left(\mathrm{CS}\cdot {\mathrm{CSF}}_{\mathrm{inh}}\cdot {\mathrm{IR}}_{\mathrm{air}}\cdot {\left(\mathrm{BW}/70\right)}^{1/3}\cdot \mathrm{EF}\cdot \mathrm{ED}\right)/\mathrm{BW}\cdot \mathrm{AT}\cdot \mathrm{PEF} $$

5 $$ \mathrm{ILCRs}={\mathrm{ILCRs}}_{\mathrm{ingestion}}+{\mathrm{ILCRs}}_{\mathrm{dernal}}+{\mathrm{ILCRs}}_{\mathrm{inhalation}} $$

For calculations of benzo(*a*)pyrene (BaP) equivalent concentrations (CS), the scheme developed by Nisbet and LaGoy (1992) was used, due to the current knowledge about toxic potency of individual PAHs relative to their BaP concentration (Petry et al. 1996), reliability and consistency across many studies (Masiol et al. 2013). It was assumed that exposed persons are working in biochar manufacturing for 25 years, 250 days per year, their average body weight is 70 kg and their life expectancy is 70 years. Conservative values of inhalation rate (15 m^3^/day) (US EPA National Center for Environmental Assessment and Washington 2011) and ingestion cancer slope factor CSF_ing_= 1.2 (mg kg^−1^ day^−1^)^−1^ were used (Gaylor et al. 2000). All the parameters used in the calculations are presented in the Supplementary material (Table S2, page 4).

## Results and discussion

It is accepted that combustion processes and release of petroleum products are the two main sources of anthropogenic PAHs in the environment (Sims and Overcash 1983). Most of those compounds accumulate in the soil (Desaules et al. 2008; Maliszewska-Kordybach et al. 2009), but precise determination of the source of their origin is not an easy task (Tobiszewski and Namieśnik 2012).

### PAH content in soils

Table 1 presents the total content of 16 PAHs in the soil samples analysed. The values of the total content fall within the range of 1.8–101.3 μg/g (median 16.5 μg/g, mean value 29.7 μg/g). The lowest levels of PAHs were observed in samples W2a (1.80 μg/g), W1c (4.20 μg/g) and W3a (6.83 μg/g). A particularly high level of those compounds was noted in sample W3b (101.28 μg/g). The level of PAHs at as many as four out of the five localities (W1a–W5b) was higher than 10 μg/g, and at two (W3b and W5b) sites, it exceeded 50 μg/g. Compared to the average levels of PAHs in the soils of Switzerland (0.145–0.593 μg/g), Germany (0.100–0.775 μg/g) (Desaules et al. 2008) and Poland (0.028–2.445 μg/g) (Maliszewska-Kordybach 1996; Oleszczuk and Pranagal 2007), those values are very high. The difference is smaller compared to the soils in the big cities of Asia. The levels of PAHs in the soils in Hong Kong fall within the range of 0.147–8.04 μg/g (average) (Man et al. 2013), while in Beijing, they vary from 0.178 to 12.14 μg/g (average), attaining a maximum level of 28.50 μg/g (Peng et al. 2011). The contents of the individual PAHs were varied and clearly depended on the sampling site. Detailed analysis of the contribution of the individual PAHs indicated the dominance of four-ring compounds in most of the samples studied (Table S3).

**Table 1:** The concentration of PAHs in examined samples, BaP_eq_ benzo(*a*)pyrene equivalent concentrations and incremental lifetime cancer risks (ILCRs)

Sample name	PAH16 (μg/g)	BaP_eq_ (μg/g)	ILCRs (–)
W1a	9.89	0.99	1.34 · 10^−3^
W1b	8.23	0.76	1.03 · 10^−3^
W1c	4.29	0.32	4.33 · 10^−4^
W2a	1.80	0.17	2.33 · 10^−4^
W2b	45.42	1.92	2.60 · 10^−3^
W3a	68.34	2.12	2.87 · 10^−3^
W3b	101.28	77.89	1.05 · 10^−1^
W4a	16.54	0.27	3.59 · 10^−4^
W4b	16.58	0.74	1.00 · 10^−3^
W5a	28.15	2.88	3.89 · 10^−3^
W5b	87.41	7.08	9.58 · 10^−3^

In accordance with the current regulations in Poland (Dz 2002), the content of the individual PAHs: naphthalene, phenanthrene, anthracene, fluoranthene, chrysene, benzo(*a*)anthracene, benzo(*a*)fluoranthene and benzo(*g*,*h*,*i*)perylene, in protected areas should be lower than 0.10 μg/g. At the same time, the highest permissible concentration of the 16 most important PAHs in soils of protected areas cannot exceed 1.00 μg/g. Similarly, strict norms are in force in the Czech Republic, Italy, Slovakia and Denmark (Carlon 2007). The warning level values in force in Germany amount to 3 μg/g at organic carbon content lower than 8 %, and 10 μg/g at organic carbon above 10 % (Desaules et al. 2008). The total concentrations in all of the samples studied considerably exceed the values permissible in Poland. The soils analysed should be considered as strongly contaminated and potentially dangerous for human health.

The high level of PAHs in the soils studied is all the more notable in view of the fact that they were sampled in an area with highly limited anthropopressure. In the vicinity of the sampling sites, there were no active industrial facilities, and there were no intensively used transport routes. At present, that area is a national park and a UNESCO biosphere reserve. We speculate that the only potential source of contamination of soils with such high PAH levels in the area is the activity related with biochar production that has been conducted here for a number of years.

### Molecular diagnostic ratios

The literature provides descriptions of more than ten different molecular diagnostic ratios (Katsoyiannis et al. 2011; Tobiszewski and Namieśnik 2012). As it was mentioned earlier, for the purpose of this study, the following MDRs were chosen: ANT/(ANT+PHE), FLA/(FLA+PYR) and IcdP/(IcdP+BghiP). The pyrogenic origin of PAHs is indicated by the values of the ratio ANT/(ANT+PHE) above 0.1 and FLA/(FLA+PYR) and IcdP/(IcdP+BghiP) higher than 0.4, with the values of the latter two exceeding the level of 0.5 being indicators of the processes of combustion of coal or biomass (grass, wood).

As mentioned before, the sole potential source of PAHs in the area under study is processes related with biochar production. To confirm that, MDRs were calculated for particular samples (Table 2). The values of the ratio ANT/(ANT+PHE) for the samples studied fall within the range of 0.172–0.807, exceeding the threshold of 0.1, characteristic for contaminants of pyrogenic origin, and thus related with biochar production. Six samples (W1b, W2a, W2b, W4a, W5a, W5b) were characterised with the ratio FLA/(FLA+PYR) above 0.5, indicating that the source of the PAHs contained in them can be grass, wood or coal combustion, and for three of the samples (W1a, W1c, W4a) that ratio is very close to 0.5 (0.495, 0.492 and 0.495, respectively). All of those values indicate clearly that the source of PAHs in the area can be processes related with biochar production.

**Table 2:** Calculated FLA/(FLA+PYR) and ANT/(ANT+PHE) ratios in examined soil samples and various biochars

Sample name	ANT/(ANT+PHE)	FLA(FLA+PYR)	Literature
W1a	0.2396	0.4951	Present work
W1b	0.1969	0.5303	
W1c	0.2139	0.4916	
W2a	0.2373	0.5568	
W2b	0.2361	0.5478	
W3a	0.8072	0.2724	
W3b	0.7758	0.1596	
W4a	0.2096	0.4959	
W4b	0.1820	0.5564	
W5a	0.1974	0.5255	
W5b	0.1719	0.5468	
grass 300 °C	0.0943	0.2293	Keiluweit et al. (2012)
grass 400 °C	0.2803	0.4263	
grass 500 °C	0.1964	0.4918	
grass 600 °C	0.0773	0.3038	
wood 300 °C	–	0.3889	
wood 400 °C	0.1006	0.3715	
wood 500 °C	0.1238	0.3302	
wood 600 °C	0.0683	0.3667	
biochar (median)	–	0.1875	Freddo et al. (2012)
biochar 1	0.1923	0.4627	Hilber et al. (2012)
biochar 2	0.1571	0.5304	
biochar 3	0.1668	0.5840	
biochar 4	0.1705	0.5495	
BC-2	0.1533	0.3230	Oleszczuk et al. (2013)
BC-W	0.1625	–	
BC-O	0.2198	0.1781	
MC-M	0.2105	–	

*–* no pyrene content

In the eight samples, the ratio of the content of indeno(*c*,*d*)pyrene and the sum of the contents of indeno(*c*,*d*)pyrene and benzo(*g*,*h*,*i*)perylene is higher than 0.5 (Table 2), which indicates that the PAHs contained in them were formed in the processes of combustion of biomass and, possibly, coal, while for sample W4a, it exceeds the value of 0.2 (0.3978) which is the threshold value for those contaminants that are the pyrogenic origin. The results obtained for those indicators also confirm that the primary source of contamination of the soils studied can be processes related with biochar production.

Samples W3a and W3b are characterised by notably lower FLA/(FLA+PYR) (0.272 and 0.160) and IcdP/(IcdP+BghiP) (0.223 and 0.086) ratios than those for the remaining samples in the series, which suggests a different origin of the PAHs. It is possible that the soil at the sampling sites was contaminated with liquid products of pyrolysis, such as creosotes. However, a simple, direct comparison with the ratio FLA/(FLA+PYR) for various creosotes, calculated on the basis of literature data (Melber et al. 2004), yielded negative results. The results of the calculations of MDR for various creosotes, presented in Table S4, fall within the range of 0.52–0.80, and thus, they are considerably higher that the values for samples W3a and W3b (Table 2). Also, the values of the ratio ANT/(ANT+PHE) for those samples (0.8017 and 0.7758) are outside of the range of values of that parameter calculated for creosotes (0.039–0.327) (Table S4). It appears, therefore, that the site from which samples W3a and W3b were collected could have been accidentally contaminated with fuel or oils from vehicles used for serving the kilns and for the transport of wood and biochar.

**Fig. 1:**
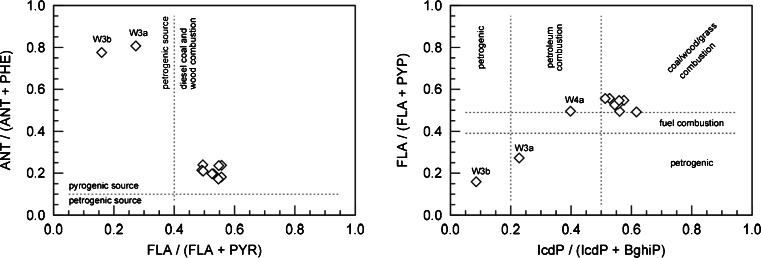
Molecular diagnostic ratios for identification of PAH pollution sources

**Fig. 2:**
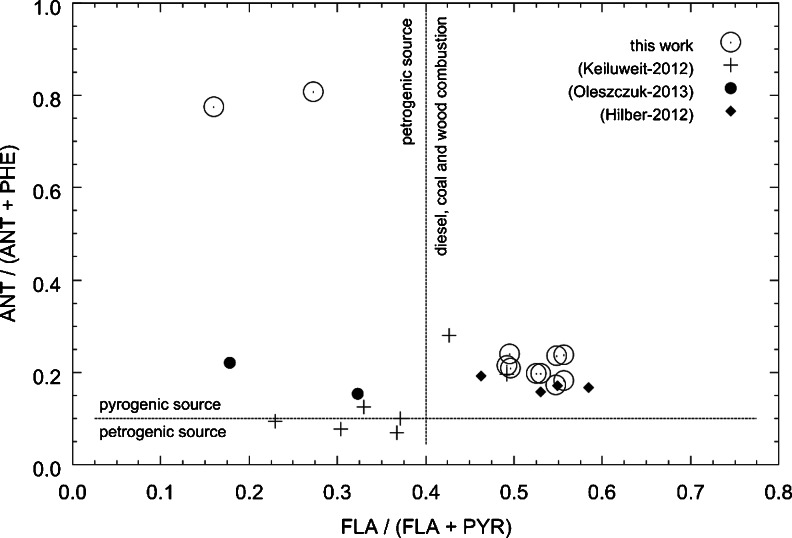
Crossplot for MDRs presented in Table 2

The results of calculations of MDRs for the soil samples studied are presented in Fig. 1 in the form of the so-called crossplots. That analysis indicates that the source of contamination of soils at the sites described is the processes of biomass combustion. Only samples W3a and W3b appear to be contaminated with hydrocarbons of petrogenic origin. Those results were additionally juxtaposed with MDRs calculated for various biochars on the basis of literature data (Fig. 2, Table 2). Out of 13 values of the ratio ANT/(ANT+PHE), nine fall within the range of 0.10–0.28, while six values of the ratio FLA/(FLA+PYR) fall in the range of 0.42–0.59, indicating considerable similarity to the soils studied, which strongly supports our thesis concerning the source of the contaminants.

Taking even several MDRs as the basis for the classification (identification) of a source may lead to misleading conclusions. Katsoyiannis et al. (2011) demonstrated than in many cases, the application of MDRs does not yield coherent results, as between the emitter and the receiver PAHs may undergo chemical transformations (interfering with the MDRs described above), and also PAHs may reach the receiver arriving from various sources.

Biochar production processes as the source of PAHs found in the samples studied are indicated, however, not only by the MDRs discussed above but also by the lack of any reports of forest fires in the area of soil sampling, by the considerable distance from intensively used transport routes and other sources of PAHs, the immediate vicinity of kilns and the very high level of those compounds in the samples.

### Incremental lifetime cancer risks

High levels of carcinogenic substances in soils may create a considerable threat to ecosystems and to human health, especially with relation to people involved in the traditional production of biochar. The most exposed individuals are the “biochar men”, working in the production of biochar throughout the season. A parameter frequently used for the description of hazard to human health is the so-called ILCRs. This unitless factor represents the increased probability of occurrence of cancer due to prolonged exposure to a toxic agent, usually taking into consideration three exposure pathways: ingestion, inhalation and dermal contact.

Following the literature data (Man et al. 2013; New York State Department of Health 2007), the ILCRs applied for the estimation of cancer risk are here classified as low (<10^−6^), medium (10^−6^–10^−4^), moderate (10^−4^–10^−3^), high (10^−3^–10^−1^) and very high (>10^−1^). The sums of the calculated ILCRs are presented in Table 1, and the full compilation (ILCRs_ingestion_, ILCRs_inhalation_, ILCRs_dermal_) is shown in Table S5 (supplemental material, page 6).

The estimated ILCRs fall within the range of 2.33 · 10^−4^ (sampleW2a)–1.05 · 10^−1^ (sample W3b). Only in the case of three samples (W1c, W2a, W4a) cancer risk can be considered to be moderate. As many as seven of the soils studied posed a high cancer risk: W1a, W1b, W2b, W3a, W4b, W5a and W5b (ILCRs from 1.00 · 10^–3^ to 9.58 · 10^−3^), and soil W3b presented a very high risk (ILCR= 1.05 · 10^−1^).

Occupational cancer risk resulting from contact with soils contaminated with PAHs was estimated by Man et. (2013). Those authors analysed soils from 55 locations in Hong Kong, under various uses (e.g. car dismantling workshops, open burning sites, e-waste open burning sites, etc.). The ILCRs values estimated by those authors fell within the range of 1.90 · 10^−7^–4.53 · 10^−4^, while the risk level was estimated as very low, low and moderate. The potential risk created by PAHs in soils within the area of Beijing was estimated by Peng et. al. (2011). The ILCRs calculated by those authors did not exceed the limit of 1.24 · 10^−4^, and one of the highest values (1.24 · 10^−3^) was measured in the vicinity of a coking plant in that city. The results obtained in this study are higher by 1 to 3 orders of magnitude, which is due primarily to the higher concentrations of PAHs. Therefore, the risk formally attributable to the calculated values of ILCRs should be considered as high and very high.

Similar to the results obtained in references Man et al. (2013), Peng et al. (2011), we also observed a relation between the dermal and ingestion and the inhalation risks, which were lower by 4 orders of magnitude (ILCRs_ingestion_ and ILCRs_dermal_>>ILCRs_inhalation_) (Table S5).

It should be emphasised that in reality, workers working with kilns have contact not only with contaminated soil but also with the biochar itself and with the fine particles formed during its production. As mentioned earlier, due to its mechanical properties, it is a much more dangerous source of dusts than the soil itself. As our analysis also does not take into account the dusts formed from biochars, the risk described should be considered as underestimated, and thus, the risk of disease among the workers may be considerably higher.

## Conclusions

The study demonstrated that soils in the immediate vicinity of kilns are strongly contaminated with PAHs. The concentrations of PAHs are considerably higher than the permissible limits laid down in the regulations in force in many countries. Both the analysed MDRs and the features of the situation of the sampling sites indicate potentially that the source of those contaminants is the production of biochar in that area. In accordance with the estimated values of ILCRs, the cancer risk resulting from contact with the contaminated soils should be considered at least high. It should be strongly emphasised that the level of the risk may be underestimated. It should also be noted that apart from the cancer risk to humans, there is a great hazard to animals in that region. The fauna of the region includes many protected species that should be granted special protection. Those sites may, therefore, create serious hazard to the ecosystems and to human health.

## Electronic supplementary material

Below is the link to the electronic supplementary material.

ESM 1 (DOCX 365 kb)
