# Supplementary material for: Biochar production increases the polycyclic aromatic hydrocarbon content in surrounding soils and potential cancer risk
Source: Environ Sci Pollut Res Int. 2013 Nov 26;21(5):3646–52. doi: 10.1007/s11356-013-2334-1 (PMC3925498; doi:10.1007/s11356-013-2334-1)
Supplement: Supplementary file 1 — (DOCX 365 kb) [file 11356_2013_2334_MOESM1_ESM.docx]

Supplementary material

**BIOCHAR PRODUCTION INCREASES THE POLYCYCLIC AROMATIC HYDROCARBONS CONTENT IN SURROUNDING SOILS AND POTENTIAL CANCER RISK**

Marcin Kuśmierz, Patryk Oleszczuk*

*Department of Environmental Chemistry, Faculty of Chemistry, 3 Maria Curie-Skłodowska Square, 20-031 Lublin, Poland*

Correspondence to Patryk Oleszczuk (phone: +48 81 5248160; fax: +48 81 5248150; e-mail: patryk.oleszczuk@poczta.umcs.lublin.pl)

Journal: Environmental Science and Pollution Research

Number of pages: 7 (including starting page)


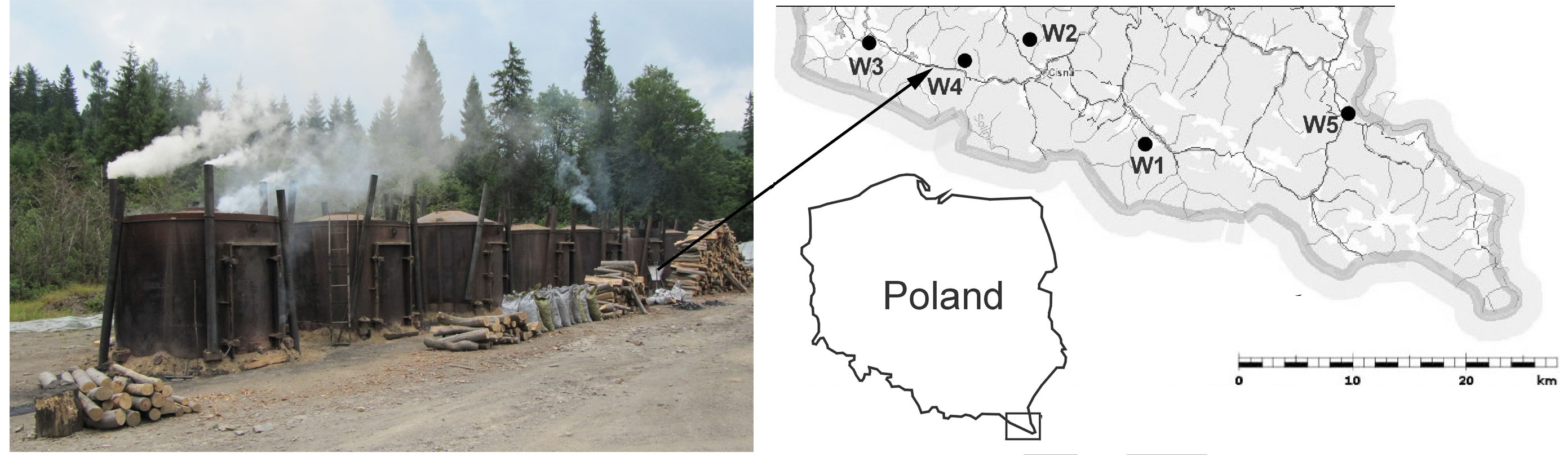


**Figure S1.** Map showing sampling sites (W1-W5) in the study area.

**Table S1**. Physicochemical parameters of investigated soils: pH - soil reaction (-), TOC - total organic carbon (wt. %), N - nitrogen (wt. %), C/N - carbon to nitrogen ratio, CEC – the cation exchange capacity (mmol/kg), Hh - hydrolytic acidity (mmol/kg).

|  | **W1a** | **W1b** | **W1c** | **W2a** | **W2b** | **W3a** | **W3b** | **W4a** | **W4b** | **W5a** | **W5b** |
| --- | --- | --- | --- | --- | --- | --- | --- | --- | --- | --- | --- |
| **pH** | 6.4 | 6.3 | 5.9 | 3.5 | 6.1 | 5.9 | 6.4 | 4.4 | 6.2 | 4.8 | 6.3 |
| **TOC** | 2.96 | 2.82 | 2.52 | 3.51 | 18.78 | 1.76 | 23.04 | 3.90 | 12.84 | 3.08 | 3.18 |
| **N** | 0.098 | 0.280 | 0.154 | 0.280 | 0.500 | 0.084 | 0.728 | 0.350 | 0.476 | 0.280 | 0.322 |
| **TOC/N** | 30.2 | 10.1 | 16.4 | 12.5 | 37.6 | 20.9 | 31.6 | 11.1 | 27.0 | 11.0 | 9.0 |
| **CEC** | 48.2 | 44.8 | 32.0 | 26.6 | 37.0 | 49.0 | 48.4 | 32.4 | 43.0 | 32.2 | 49.4 |
| **Hh** | 0.375 | 0.300 | 0.450 | 7.875 | 0.750 | 0.300 | 0.600 | 4.650 | 0.525 | 2.850 | 0.450 |

**Table S2**. Equations and parameters used for estimating ICLRS (Budroe et al., 2009; Gaylor et al., 2000; Knafla et al., 2006; Peng et al., 2011; US EPA, 2011; US EPA OSWER, 2009, 1991).

| **Exposure factors** | **symbol** | **value** | **unit** |
| --- | --- | --- | --- |
| benzo(a)pyrene equivalent concentration  calculated as a sum of individual PAH concentrations multiplied by corresponding TEF factor (table S3):  C_NAP_∙TEF_NAP_ + … + C_IcdP_∙TEF_IcdP_ | CS | see table 1 | mg/kg |
| conversion factor | CF | 10^-6^ | kg/mg |
| carcinogenic slope factor (inhalation)* | CSF_inh_ | 3.9 | (mg∙kg^-1^∙day^-1^)^-1^ |
| carcinogenic slope factor (ingestion)*** | CSF_ing_ | 1.2 | (mg∙kg^-1^∙day^-1^)^-1^ |
| carcinogenic slope factor (dermal)** | CSF_der_ | 25.00 | (mg∙kg^-1^∙day^-1^)^-1^ |
| body weight | BW | 70 | kg |
| average life span (70 years ∙ 365 day∙year^-1^) | AT | 22550 | day |
| soil intake rate | IR_soil_ | 20 | mg∙day^-1^ |
| inhalation rate | IR_air_ | 15 | m^3^∙day^-1^ |
| dermal surface exposure (face and hands) | SA | 1423 | cm^2^ |
| dermal adherence factor (gardeners, face, forearms, hands) | AF | 0.102 | mg∙cm^-2^ |
| dermal adsorption fraction | ABS | 0.1 | - |
| soil dust produce factor**** | PEF | 1.36∙10^9^ | m^3^∙kg^-1^ |
| event frequency | EV | 1 | day^-1^ |
| exposure frequency | EF | 250 | day∙year^-1^ |
| exposure duration | ED | 25 | year |

*(Budroe et al., 2009) **(Knafla et al., 2006) ***(Gaylor et al., 2000) ****(Peng et al., 2011)

**Table S3**. PAHs concentrations in examined samples (µg/g) and PAHs toxic equivalency factors with respect to benzo(a)pyrene (µg/g) (Collins et al., 1998; Nisbet and LaGoy, 1992).

| **PAH** | **W1a** | **W1b** | **W1c** | **W2a** | **W2b** | **W3a** | **W3b** | **W4a** | **W4b** | **W5a** | **W5b** | **TEF** |
| --- | --- | --- | --- | --- | --- | --- | --- | --- | --- | --- | --- | --- |
| NAP | <LOD | <LOD | <LOD | <LOD | 1.08 | <LOD | <LOD | <LOD | <LOD | <LOD | <LOD | 0.001 |
| ACY | <LOD | <LOD | <LOD | <LOD | 2.10 | <LOD | <LOD | <LOD | <LOD | <LOD | <LOD | 0.001 |
| ACE | 1.53 | 2.73 | <LOD | <LOD | 7.37 | 1.13 | 4.03 | 9.07 | <LOD | <LOD | <LOD | 0.001 |
| FLO | 0.20 | 0.12 | 0.29 | 0.13 | 2.06 | 0.04 | 0.12 | 0.42 | 0.75 | 0.66 | 1.62 | 0.001 |
| PHE | 0.87 | 0.29 | 0.65 | 0.24 | 6.87 | 0.01 | 0.05 | 1.40 | 3.58 | 3.65 | 10.78 | 0.001 |
| ANT | 0.27 | 0.07 | 0.18 | 0.08 | 2.12 | 0.05 | 0.19 | 0.37 | 0.80 | 0.90 | 2.24 | 0.01 |
| FLA | 1.77 | 1.27 | 0.87 | 0.42 | 8.75 | 0.47 | 0.56 | 1.72 | 4.38 | 6.43 | 24.14 | 0.001 |
| PYR | 1.80 | 1.12 | 0.90 | 0.34 | 7.22 | 1.26 | 2.95 | 1.75 | 3.49 | 5.81 | 20.01 | 0.001 |
| BaA | 0.73 | 0.48 | 0.36 | 0.09 | 2.04 | 0.68 | 3.12 | 0.58 | 0.91 | 2.20 | 6.83 | 0.1 |
| CHR | 0.70 | 0.51 | 0.32 | 0.11 | 1.93 | 0.31 | 1.26 | 0.61 | 1.10 | 2.26 | 6.79 | 0.01 |
| BbF | 0.56 | 0.49 | 0.26 | 0.12 | 1.24 | 0.28 | 2.62 | 0.32 | 0.55 | 1.81 | 4.67 | 0.1 |
| BkF | 0.28 | 0.24 | 0.13 | 0.06 | 0.61 | 0.27 | 4.68 | 0.12 | 0.21 | 0.91 | 2.42 | 0.1 |
| BaP | 0.48 | 0.35 | 0.15 | 0.06 | 0.94 | 0.29 | 8.31 | 0.10 | 0.35 | 1.44 | 3.67 | 1 |
| DahA | 0.32 | 0.26 | 0.08 | 0.08 | 0.48 | 1.69 | 68.43 | 0.03 | 0.18 | 0.82 | 1.73 | 1 |
| BghiP | 0.16 | 0.13 | 0.04 | 0.03 | 0.25 | 0.26 | 4.55 | 0.03 | 0.14 | 0.58 | 1.11 | 0.01 |
| IcdP | 0.21 | 0.17 | 0.06 | 0.04 | 0.33 | 0.08 | 0.43 | 0.02 | 0.14 | 0.69 | 1.41 | 0.1 |
| PAH16 | 9.89 | 8.23 | 4.29 | 1.80 | 45.42 | 6.83 | 101.28 | 16.54 | 16.58 | 28.15 | 87.41 | NA |

NAP – naphtalene, ACY – acenaphtylene, ACE – acenaphtene, FLO – fluorene,
PHE – phenantrene, ANT – anthracene, FLA – fluoranthene, PYR – pyrene,
BaA – benzo(a)anthracene, CHR – chrysene, BbF – benzo(b)fluoranthene,
BkF – benzo(k)fluoranthene, BaP –  benzo(a)pyrene, DahA – dibenzo(ah)anthracene,
BghiP – benzo(ghi)perylene, IcdP – indeno(cd)pyrene,
TEF – toxic equivalency factor with respect to benzo(a)pyrene

**Table S4**. Calculated FLA/(FLA+PYR) and ANT/(ANT+PHE) ratios in coal tar creosotes, and creosotes used in environmental/toxicological studies, based on data from (Melber et al., 2004).

| **sample name  / page** | **FLA/(FLA+PYR)** | **ANT/(ANT+PHE)** |
| --- | --- | --- |
| (A) / 14 | 0.5205 / 0.5405 | 0.0869 |
| (B) / 14 | 0.6071 | - |
| (D) / 14 | 0.5869 | 0.3267 |
| (E) / 14 | 0.5542 | 0.1318 |
| (F) / 14 | 0.6880 | 0.0810 |
| (G) / 14 | 0.6676 | 0.1067 |
| Lehto  / 15 | 0.6804 | 0.2076 |
| Bestari / 15 | 0.5454 | 0.0543 |
| CPT1 / 15 | 0.8000 | 0.0882 |
| CPT2 / 15 | 0.6406 | 0.0391 |
| median | 0.6071 | 0.0882 |
| mean | 0.6199 | 0.1211 |

**Table S5**. Calculated incremental lifetime cancer risks (ILCRS).

| **sample** | **W1a** | **W1b** | **W1c** | **W2a** | **W2b** | **W3a** | **W3b** | **W4a** | **W4b** | **W5a** | **W5b** |
| --- | --- | --- | --- | --- | --- | --- | --- | --- | --- | --- | --- |
| **ILCRS_inhalation_** | 1.49∙10^-7^ | 1.14∙1^-7^ | 4.81∙10^-8^ | 2.59∙10^-8^ | 2.89∙10^-7^ | 3.19∙10^-7^ | 1.17 ∙10^-5^ | 3.99∙10^-8^ | 1.11∙10^-7^ | 4.32∙10^-7^ | 1.06∙10^-6^ |
| **ILCRS_ingestion_** | 8.33∙10^-5^ | 6.38∙10^-5^ | 2.68∙10^-5^ | 1.45∙10^-5^ | 1.61∙10^-4^ | 1.78∙10^-4^ | 6.53∙10^-3^ | 2.23∙10^-5^ | 6.21∙10^-5^ | 2.41∙10^-4^ | 5.94∙10^-4^ |
| **ILCRS_dermal_** | 1.26∙10^-3^ | 9.64∙10^-4^ | 4.06∙10^-4^ | 2.19∙10^-4^ | 2.44∙10^-3^ | 2.69∙10^-3^ | 9.88∙10^-2^ | 3.37∙10^-4^ | 9.39∙10^-4^ | 3.65∙10^-3^ | 8.98∙10^-3^ |
| **ILCRS** | 1.34∙10^-3^ | 1.03∙10^-3^ | 4.33∙10^-4^ | 2.33∙10^-4^ | 2.60∙10^-3^ | 2.87∙10^-3^ | 1.05∙10^-1^ | 3.59∙10^-4^ | 1.00∙10^-3^ | 3.89∙10^-3^ | 9.58∙10^-3^ |

**REFERENCES**

Budroe, J.D., Brown, J.P., Collins, J.F., Marty, M.A., Salmon, A.G., Sandy, M.S., Sherman, C.D., Tomar, R.S., Zeise, L., 2009. Technical Support Document for Cancer Potency Factors. Appendix H [WWW document]. URL http://www.oehha.ca.gov/air/hot_spots/2009/TSDCancerPotency.pdf. (accessed 10.9.13)

Collins, J.F., Brown, J.P., Alexeeff, G.V., Salmon, A.G., 1998. Potency Equivalency Factors for Some Polycyclic Aromatic Hydrocarbons and Polycyclic Aromatic Hydrocarbon Derivatives. Regulatory Toxicology and Pharmacology 28, 45–54.

Gaylor, D.W., Culp, S.J., Goldstein, L.S., Beland, F.A., 2000. Cancer Risk Estimation for Mixtures of Coal Tars and Benzo(a)pyrene. Risk Analysis 20, 81–86.

Knafla, A., Phillipps, K.A., Brecher, R.W., Petrovic, S., Richardson, M., 2006. Development of a dermal cancer slope factor for benzo[a]pyrene. Regulatory Toxicology and Pharmacology 45, 159–168.

Melber, C., Kielhorn, J., Mangelsdorf, I., Organization, W.H., Safety, I.P. on C., 2004. Coal tar creosote [WWW Document]. URL http://apps.who.int/iris/handle/10665/42943 (accessed 10.9.13).

Nisbet, I.C.T., LaGoy, P.K., 1992. Toxic equivalency factors (TEFs) for polycyclic aromatic hydrocarbons (PAHs). Regulatory Toxicology and Pharmacology 16, 290–300.

Peng, C., Chen, W., Liao, X., Wang, M., Ouyang, Z., Jiao, W., Bai, Y., 2011. Polycyclic aromatic hydrocarbons in urban soils of Beijing: Status, sources, distribution and potential risk. Environmental Pollution 159, 802–808.

US EPA, 2011. Exposure Factors Handbook 2011 Edition (Final) [WWW Document]. URL http://cfpub.epa.gov/ncea/risk/recordisplay.cfm?deid=236252 (accessed 10.9.13).

US EPA OSWER, 1991. Risk Assessment Guidance for Superfund (RAGS) Part B [WWW Document]. URL http://www.epa.gov/oswer/riskassessment/ragsb/index.htm (accessed 10.9.13).

US EPA OSWER, 2009. Risk Assessment Guidance for Superfund (RAGS), Volume I: Human Health Evaluation Manual (Part E, Supplemental Guidance for Dermal Risk Assessment) Interim [WWW Document]. URL http://www.epa.gov/oswer/riskassessment/ragse/index.htm (accessed 10.9.13).
